# Enhanced Absorption Dominated Electromagnetic Interference Shielding Enabled by Carbon Nanotube and Graphene Reinforced Electrospun PVDF Nanocomposite

**DOI:** 10.3390/polym18070789

**Published:** 2026-03-25

**Authors:** Hisham Bamufleh, Usman Saeed, Abdulrahim Alzahrani, Aqeel Ahmad Taimoor, Sami-ullah Rather, Hesham Alhumade, Walid M. Alalayah, Hamad AlTuraif

**Affiliations:** Chemical & Materials Engineering Department, Faculty of Engineering, King Abdulaziz University, Jeddah 21589, Saudi Arabiaaataimoor@kau.edu.sa (A.A.T.);

**Keywords:** graphene, carbon nanotubes (CNT), polyvinylidene fluoride (PVDF), electromagnetic interference shielding, composites

## Abstract

The increasing density of wireless and wearable electronic devices necessitates the development of lightweight, flexible, and absorption-dominated electromagnetic interference (EMI) shielding materials. In this study, electrospun poly(vinylidene fluoride) (PVDF) composite mats reinforced with carbon nanotubes (CNTs) and graphene nanosheets at low filler loadings (1–3 wt.%) were fabricated and systematically investigated for X-band (8.0–12.5 GHz) EMI shielding performance. Raman, FTIR, and thermal analyses confirm enhanced electroactive β-phase formation and improved thermal stability upon nanofiller incorporation. The formation of interconnected conductive networks within the electrospun fibrous architecture leads to a significant increase in electrical conductivity from 10^−7^ S·cm^−1^ for pure PVDF to 10^−2^ S·cm^−1^ and 10^−1^ S·cm^−1^ for CNT/PVDF and Graphene/PVDF composites, respectively, at 3 wt.% loading. Consequently, the total EMI shielding effectiveness (SE_T_) increases from 2.5 dB for pure PVDF to 40 dB for CNT/PVDF and 42 dB for graphene/PVDF composites at 3 wt.%. The shielding effectiveness arising from absorption (SE_A_) dominates the overall EMI shielding performance, contributing more than 85% of the total shielding effectiveness (SE_T_), which clearly indicates an absorption-controlled shielding mechanism. The combination of high absorption-dominated EMI shielding, low filler content, and mechanical flexibility highlights these electrospun CNT/PVDF and graphene/PVDF composites as promising candidates for next-generation flexible, wearable, and biomedical EMI shielding applications.

## 1. Introduction

The rapid advancement of electronic technologies, wireless communication systems, and miniaturized electronic devices has significantly increased electromagnetic pollution in modern environments. Electromagnetic interference (EMI) generated by electronic components, communication networks, and power transmission systems can adversely affect the performance, reliability, and lifespan of electronic devices [1,2,3,4]. More critically, EMI poses serious risks in biomedical and healthcare environments, where interference may disrupt the functioning of sensitive diagnostic equipment, implantable medical devices, and wearable electronics. Consequently, the development of efficient EMI shielding materials that are lightweight, flexible, and biocompatible has become a pressing research priority [5,6,7]. Traditionally, metals such as copper, aluminum, and steel have been widely used for EMI shielding due to their high electrical conductivity and reflection-dominated shielding mechanisms [8,9]. However, metallic shields suffer from several intrinsic drawbacks, including high density, susceptibility to corrosion, poor flexibility, and limited design adaptability [10]. These limitations make metallic materials unsuitable for emerging applications such as wearable electronics, flexible biomedical devices, and implant-adjacent systems. In contrast, polymer-based EMI shielding materials offer advantages including low weight, corrosion resistance, mechanical flexibility, and facile processability [11,12,13]. Despite these benefits, most polymers are electrically insulating and therefore require functional modification to achieve effective EMI shielding performance [14].

Poly(vinylidene fluoride) (PVDF) has emerged as an attractive polymer matrix for advanced functional composites owing to its excellent chemical resistance, thermal stability, mechanical durability, and inherent biocompatibility [15,16,17]. PVDF also exhibits unique piezoelectric and dielectric properties, which are advantageous for multifunctional electronic and biomedical applications [18,19,20]. Nevertheless, pristine PVDF lacks sufficient electrical conductivity, resulting in negligible EMI shielding effectiveness. To address this limitation, the incorporation of electrically conductive fillers into the PVDF matrix has been widely explored as an effective strategy to impart EMI shielding functionality [21]. Among various conductive fillers, carbon nanotubes (CNTs) and graphene have attracted considerable attention due to their outstanding electrical, thermal, and mechanical properties. CNTs possess a one-dimensional structure with extremely high aspect ratios, which facilitates the formation of percolated conductive networks at relatively low filler loadings [22,23,24,25]. This characteristic is particularly beneficial for maintaining mechanical flexibility and minimizing filler-induced brittleness. Graphene, a two-dimensional carbon nanomaterial, exhibits exceptional electrical conductivity, large specific surface area, and strong interfacial interactions with polymer matrices [26,27,28,29]. The planar structure of graphene promotes enhanced interfacial polarization and dielectric loss, contributing to efficient absorption-dominated EMI shielding. In PVDF-based composites, CNTs and graphene not only enhance electrical conductivity but also improve thermal stability and mechanical reinforcement through effective stress transfer and heat dissipation pathways [30]. Electromagnetic shielding effectiveness in polymer nanocomposites is governed by multiple mechanisms, including reflection, absorption, and multiple internal reflections. While reflection dominates in metal-based shields, absorption-driven EMI shielding is preferred for biomedical and wearable applications to minimize secondary electromagnetic pollution [31]. Conductive nanofillers embedded within polymer matrices promote absorption through ohmic losses, interfacial polarization, and dipolar relaxation. Additionally, the formation of interconnected conductive networks increases the attenuation of electromagnetic waves by extending their propagation path within the material [32,33,34].

Electrospinning has emerged as a versatile and scalable technique for fabricating polymer nanofibers with controlled morphology, high surface area-to-volume ratios, and interconnected porous architectures [33,34,35]. Electrospun nanofibrous mats offer distinct advantages for EMI shielding applications, including enhanced electromagnetic wave scattering, multiple reflections, and efficient energy dissipation within the fibrous network. The high porosity and large surface area of electrospun mats significantly increase the interaction between electromagnetic waves and conductive fillers, thereby improving absorption-dominated shielding performance [36,37]. Furthermore, electrospun PVDF nanofibers exhibit excellent flexibility, breathability, and conformability, making them particularly suitable for wearable and biomedical EMI shielding applications. In addition to EMI shielding performance, materials intended for biomedical use must exhibit adequate thermal stability, surface wettability, mechanical integrity, and biocompatibility. Thermal stability is essential for maintaining performance under operational and sterilization conditions, while surface wettability plays a critical role in cell adhesion and proliferation. PVDF-based composites reinforced with CNTs and graphene have demonstrated tunable surface energy and enhanced thermal resistance; however, excessive filler loading may lead to agglomeration, compromised mechanical properties, and potential cytotoxic effects [38,39]. Therefore, optimizing filler content and ensuring homogeneous dispersion within the polymer matrix are crucial for achieving balanced multifunctional performance. Recent studies have demonstrated the potential of CNT and graphene-reinforced polymer composites for EMI shielding; however, comprehensive investigations combining electrospun nanofibrous architecture, low filler loading, absorption-dominated shielding, and biocompatibility remain limited [40,41,42]. In particular, systematic comparisons between CNT/PVDF and Graphene/PVDF electrospun mats with controlled filler concentrations are scarce. Understanding the structure–property relationships governing conductive network formation, electromagnetic attenuation mechanisms, and biological response is essential for the rational design of next-generation EMI shielding materials for biomedical applications [43].

In this work, electrospun nanofibrous mats of pure PVDF, CNT/PVDF, and Graphene/PVDF composites with varying filler loadings (1, 2, and 3 wt.%) are developed and systematically investigated. The influence of filler type and concentration on morphological characteristics, electrical conductivity, thermal behavior and electromagnetic shielding effectiveness in the X-band frequency range (8.0–12.5 GHz) is comprehensively evaluated. Special emphasis is placed on distinguishing the EMI shielding contributions arising from absorption (SE_A_) and reflection (SE_R_) mechanisms. The results demonstrate that electrospun CNT/PVDF and Graphene/PVDF nanofibrous mats achieve significantly enhanced EMI shielding effectiveness while retaining mechanical flexibility. Furthermore, the formation of conductive networks within the PVDF matrix significantly improves the shielding effectiveness. These findings demonstrate the potential of the developed nanocomposites for lightweight and flexible EMI shielding applications.

## 2. Materials and Methods

### 2.1. Materials

PVDF (6060D) with a density of 1.24 g/cm^3^ and a melt flow rate of 5.76 g/10 min was purchased from Alfa Aesar GMBH (Karlsruhe, Germany) and multi-walled CNTs with a diameter of 10–20 nm, length of 10–30 µm, were bought from Beijing Tiannai Co., Ltd. (Beijing, China). C750-grade graphene nanoplatelets (GNPs) supplied by Sigma Aldrich (Vienna, Austria) were employed as reinforcement fillers having an average particle size of 2 μm and a specific surface area of about 750 m^2^ g^−1^, while dimethylformamide (DMF 99.5%) was obtained from Taicang Shanghai Test Reagent Co., Ltd., Shanghai, China.

### 2.2. Preparation of CNT/PVDF and Graphene/PVDF Composites

Poly(vinylidene fluoride) (PVDF)-based nanocomposites reinforced with carbon nanotubes (CNTs) and graphene were prepared using a solution blending approach followed by electrospinning to obtain nanofibrous mats. Initially, PVDF was dissolved in a mixed solvent system consisting of dimethylformamide (DMF) and acetone in a volumetric ratio of 6:5. The polymer solution was prepared by adding PVDF to the solvent mixture to obtain a total polymer concentration of 10 wt.%. The solution was magnetically stirred at 60 °C for 6–8 h until a clear and homogeneous solution was obtained, ensuring complete dissolution of PVDF.

For the preparation of MWCNT/PVDF composites, MWCNTs were added to the PVDF solution at different weight fractions of 1, 2, and 3 wt.% with respect to the PVDF. The CNT dispersion was then slowly introduced into the PVDF solution under continuous stirring, followed by further ultrasonication for 30 min and magnetic stirring for 4–6 h to obtain a stable and homogeneous CNT/PVDF spinning solution. Similarly, the graphene was subsequently added to the PVDF solution at loadings of 1, 2, and 3 wt.% relative to wt.% of PVDF. The resulting mixtures were stirred and sonicated using the same protocol as for MWCNT/PVDF composites to ensure uniform dispersion.

The prepared composite solutions were subjected to electrospinning to fabricate nanofibrous mats. Electrospinning was carried out using a syringe pump (TL BM, TONG TL, Shenzhen, China) equipped with a stainless-steel needle and a flat aluminum collector was used to collect the electrospun fibers. The optimized electrospinning parameters included an applied voltage of 15–20 kV, a solution flow rate of 0.8 mL·h^−1^ and a tip-to-collector distance of 15–18 cm. In addition, the environmental conditions during electrospinning were carefully monitored and maintained at an ambient temperature of 25 ± 2 °C and a relative humidity of 55 ± 5%. These parameters were kept consistent throughout the fabrication process to ensure uniform fiber formation and reproducible nanofiber morphology. Table 1 shows the electrospun parameters.

**Figure 1:**
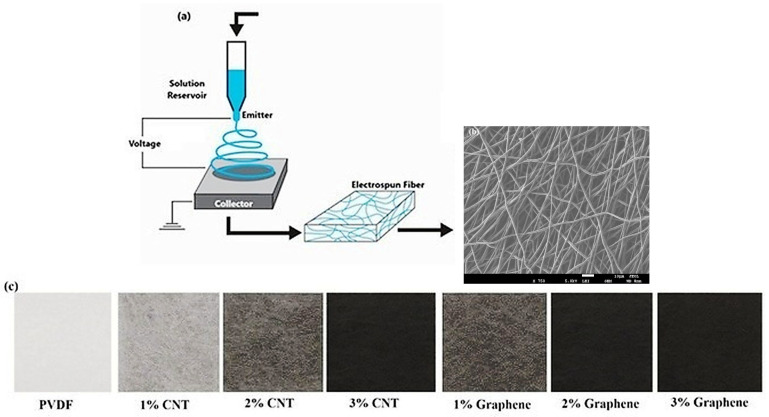
(**a**) Schematic representation of the electrospinning process, (**b**) SEM image of electrospun fiber, (**c**) optical image of the fabricated electrospun PVDF nanofibrous mat.

The electrospun mats were vacuum-dried at 40 °C for 24 h to remove residual solvents prior to characterization. All electrospinning experiments were conducted at ambient temperature and relative humidity of 40–50%. Figure 1 shows the schematic representation of the electrospinning process with SEM and a digital image of the developed electrospun mat.

### 2.3. Characterization

The surface morphology and elemental composition of the electrospun fibers were examined using scanning electron microscopy (SEM, JEOL 7600F, Tokyo, Japan) at 20 KV.

The chemical structure of the CNT/PVDF and Graphene/PVDF composite was analyzed using FTIR spectroscopy (Thermo Scientific Nicolet 6700, Waltham, MA, USA), with spectra collected in the 400–4000 cm^−1^ region.

Raman spectra were acquired using a LabRAM HR800 Raman spectrometer (Horiba Jobin Yvon, Palaiseau, France) with an excitation wavelength of 633 nm.

The thermal behavior of the PVDF/MWCNT and graphene/CNT samples was analyzed using a differential scanning calorimeter (DSC, Netzsch 200 F3, Selb, Germany). Heating and cooling cycles were conducted from 20 to 180 °C at a rate of 10 °C min^−1^. The resulting thermograms were used to determine the melting temperature, melting enthalpy, crystallization temperature, and degree of crystallinity (Xc%).

Thermogravimetric analysis (TGA) was performed using a Setaram Setsys Evolution 1750 instrument (SETARAM, KEP Technologies, Caluire-et-Cuire, France). Approximately 5 mg of sample was heated from 30 to 600 °C at a heating rate of 10 °C min^−1^ under an inert argon atmosphere. The variations in sample mass and temperature were recorded as a function of temperature, and derivative thermogravimetric (DTG) curves were used to identify the thermal degradation stages.

Tensile testing of the electrospun MWCNT/PVDF composites was performed using an Instron 1122 universal testing machine (Buckinghamshire, UK) with a crosshead speed of 1 mm min^−1^. Rectangular specimens measuring approximately 40 mm × 6 mm were prepared from the electrospun fiber mats.

Electrical conductivity characterization was performed using a four-point probe system (Ossila, Sheffield, UK), with applied voltages of 100 µV–10 V and currents of 10 nA–100 mA. For samples with thicknesses of 0.3–0.4 mm, sheet resistance was measured at three separate points and used to calculate conductivity following Equation (1).

(1) $$\sigma =\frac{1}{R_{s}t}$$

In the equation, *σ* is the electrical conductivity (S/cm), *R_s_* (Ω per square) is the sheet resistance and t is the sample thickness (cm). The electrical conductivity measurements were performed using three independent samples for each composition, and the reported values represent the mean conductivity with the corresponding standard deviation.

The electromagnetic interference (EMI) shielding performance of the MWCNT and graphene-reinforced PVDF composites was evaluated using a vector network analyzer (VNA, Agilent E5071C, Santa Clara, CA, USA) equipped with a rectangular waveguide operating in the X-band frequency range (8.2–12.4 GHz). Standard composite specimens with dimensions of 14 mm × 2 mm were prepared and mounted in the waveguide sample holder for measurement. EMI measurements were conducted on three independently prepared samples for each composition to ensure reproducibility. Scattering parameters (S_11_ and S_21_) were recorded across the X-band, and the total shielding effectiveness (SE_t_), as well as the contributions from absorption (SE_a_) and reflection (SE_r_), were calculated accordingly. The EMI shielding behavior was further correlated with electrical conductivity, nanofiller content, and microstructural characteristics to elucidate the dominant shielding mechanisms.

## 3. Results and Discussions

### 3.1. Microstructure

**Figure 2:**
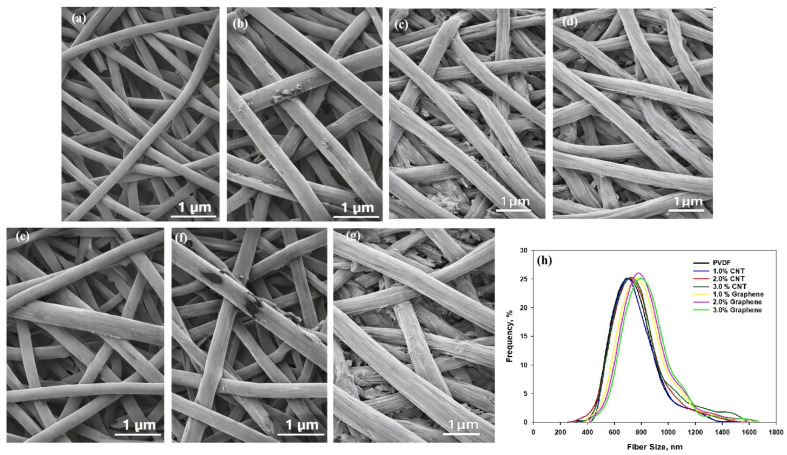
SEM of electrospun MWCNT/PVDF and Graphene/PVDF fibers. (**a**) PVDF, (**b**) 1% CNT, (**c**) 2% CNT, (**d**) 3% CNT, (**e**) 1% graphene, (**f**) 2% graphene, (**g**) 3% graphene. (**h**) Fiber size.

Figure 2 shows the surface morphology and structural features of the electrospun PVDF, CNT/PVDF, and Graphene/PVDF composite fibers. Also, Figure 2h shows the fiber size distribution. The micrographs reveal that the pure PVDF nanofibers exhibit a uniform, bead-free morphology with smooth surfaces and an average fiber diameter of approximately 600 ± 50 nm. This indicates a stable electrospinning process and good chain entanglement of PVDF, leading to continuous and well-formed fibers. Upon the incorporation of CNTs, the fiber morphology undergoes a progressive change. At 1 wt.% CNT, the fibers maintain a smooth and uniform morphology with an average diameter of approximately 700 ± 40 nm, indicating that the enhanced electrical conductivity of the spinning solution facilitates more consistent fiber formation. At 2 wt.% CNT, the average diameter further increases to 720 ± 35 nm, and the fibers exhibit mild surface roughness due to the partial exposure of CNTs. At 3 wt.% CNT, localized aggregation of CNTs becomes visible, resulting in slightly uneven surfaces and an average diameter of 760 ± 40 nm. The presence of interconnected CNT networks along and across fibers is evident, which is beneficial for charge transport and electromagnetic shielding efficiency. For Graphene/PVDF composites, the morphological features differ due to the planar geometry of graphene sheets. At 1 wt.% graphene, the fibers are smooth and uniform with an average diameter of 770 ± 45 nm, which is similar to pristine PVDF. Increasing the graphene content to 2 wt.% results in slightly flattened fibers with an average diameter of 810 ± 40 nm. At 3 wt.% graphene, the fibers having an average diameter of 890 ± 35 nm exhibit rougher surfaces, suggesting the presence of overlapping graphene flakes. CNT-filled PVDF fibers tend to exhibit larger fiber diameters, which contribute to enhanced electrical conductivity through the formation of more effective conductive pathways during electrospinning. In contrast, graphene-filled PVDF fibers show diameter broadening and surface roughening at higher loadings due to increased solution viscosity and platelet aggregation. These morphological differences directly reflect the distinct geometry and dispersion behavior of CNTs versus graphene within the electrospun PVDF matrix. Furthermore, the uniform morphology and well-dispersed conductive fillers observed in the SEM micrographs of the electrospun fibers indicate that the suspensions maintained good stability throughout the electrospinning process.

### 3.2. Chemical and Structural Analysis

**Figure 3:**
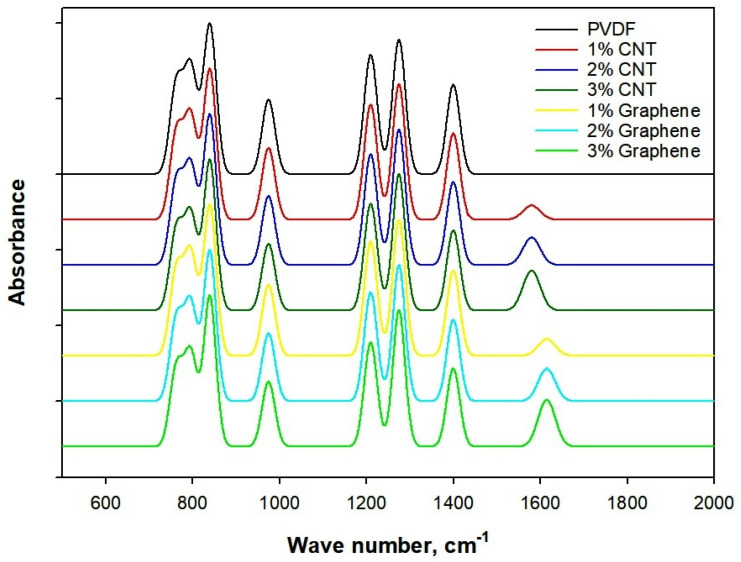
FTIR spectrum of CNT/PVDF and Graphene/PVDF nanocomposite.

Figure 3 reveals the FTIR spectrum of electrospun CNT/PVDF and Graphene/PVDF composite. The FTIR spectra of pure PVDF exhibit characteristic absorption bands at 1402 and 1200 cm^−1^ corresponding to CH_2_ bending and CF_2_ stretching vibrations, confirming the semi-crystalline nature of PVDF. The strong peaks observed at 840 cm^−1^ and 1275 cm^−1^ are attributed to the electroactive β-phase corresponding to CF_2_ stretching and CH_2_ wagging vibrations, which is highly desirable for electrical and electromagnetic applications [44]. Also, the α-phase is identified by absorption peaks at 795 cm^−1^ and 980 cm^−1^ arising from CF_2_ bending and skeletal vibrations. To quantitatively evaluate the crystalline phase composition of PVDF, the fraction of β-phase was calculated from the FTIR spectra using the absorbance intensities [43]. The β-phase fraction was determined using Equation (2).

(2) $$F\left(\beta \right)=\frac{A_{\beta }}{K_{\beta }+K_{\alpha }(A_{\alpha }+A_{\beta })}\times 100$$

where *A_β_* and *A_α_* correspond to absorbance at 840 cm^−1^ for β-phase and absorbance at 795 cm^−1^ for α-phase. Also, *K_β_* = 7.7 × 10^4^ cm^2^/mol and *K_α_* = 6.1 × 10^4^ cm^2^/mol are the absorption coefficients for β and α phases.

The neat PVDF exhibited absorbance values of 0.42 and 0.55 at 795 cm^−1^ (α-phase) and 840 cm^−1^ (β-phase), respectively, corresponding to a β-phase fraction of approximately 62%. This indicates that electrospinning promotes partial transformation of the nonpolar α-phase into the electroactive β-phase.

Upon incorporation of CNTs and graphene, a weak C=C stretching vibration associated with *sp*^2^-hybridized carbon domains appears in the range of 1580–1610 cm^−1^, confirming the presence of carbon nanofillers, indicating their physical embedding and good interfacial integration within the PVDF matrix without the formation of new chemical bonds. However, a noticeable increase in intensity of the β-phase bands is observed from 62% to 68% with increasing CNT and graphene loading, particularly at 2 and 3 wt.%. This enhancement can be attributed to strong interfacial interactions between the carbon nanomaterials and PVDF chains, which facilitate dipole alignment and promote the α-to-β-phase transformation during the electrospinning process. Additionally, the slight broadening of the CF_2_ stretching peaks in CNT/PVDF and Graphene/PVDF composites suggests restricted polymer chain mobility due to filler polymer interactions [45]. The absence of peak shifts or degradation-related bands confirms that the electrospinning process and filler incorporation do not chemically degrade PVDF. FTIR results demonstrate successful composite formation and filler-induced enhancement of the electroactive phase, which directly contributes to improved electrical conductivity and electromagnetic shielding performance.

**Figure 4:**
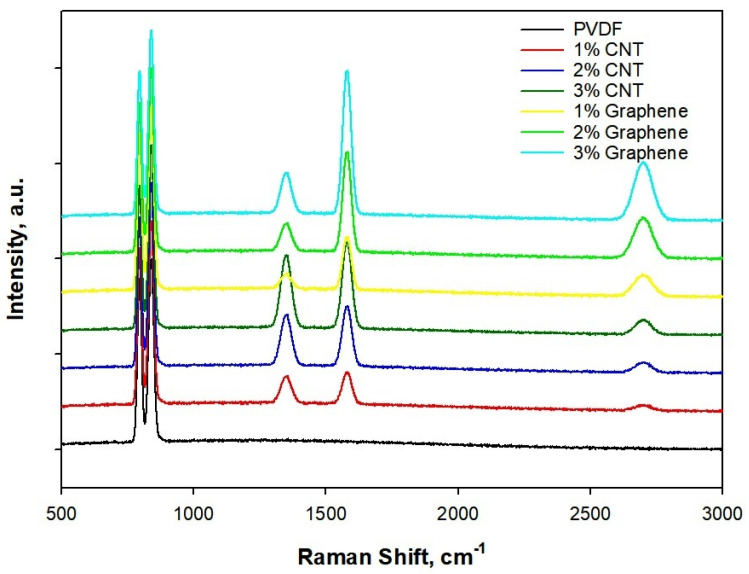
Raman spectra of CNT/PVDF and Graphene/PVDF nanocomposite.

Raman spectra, Figure 4, of electrospun PVDF and PVDF nanocomposites with CNT and graphene (1–3 wt.%) reveal clear signatures of filler incorporation, polymer phase evolution and filler matrix interactions. Pure electrospun PVDF shows the characteristic vibrational bands associated with its semi-crystalline phases, most notably the strong bands near 796 cm^−1^ and 842 cm^−1^, corresponding to the *α*- and *β*-phase chain conformations, respectively. Concomitantly, the PVDF β-phase marker at 842 cm^−1^ shows a small increase in relative intensity for both CNT and graphene composites, consistent with filler-induced nucleation and chain alignment during electrospinning. Introduction of CNTs and graphene produces additional intense features in the 1350 cm^−1^ (D-band) and 1580 cm^−1^ (G-band) regions, with the G band becoming progressively pronounced as filler loading increases [46]. For CNT/PVDF composites, the D/G intensity ratio is moderate and remains roughly constant with low loading (1 wt.%), while at higher loadings (2–3 wt.%) the G-band sharpens and weak D-band broadening indicates improved graphitic contact and limited structural disorder introduced during processing. Graphene/PVDF composites show a strong, relatively sharp G band and a smaller D band at low loadings, with D/G intensity ratio increasing slightly at 3 wt.% due to edge defects and partial restacking. The 2D band peak appears at 2700 cm^−1,^ which is discernible for graphene-rich samples and gains intensity with loading, confirming a multilayer graphitic domain in comparison to CNT. Raman data confirm successful incorporation and percolation of conductive fillers and indicate filler-assisted β-phase promotion in PVDF, a synergistic effect that helps to explain the observed enhancements in dielectric response, electrical conductivity, and EMI shielding [44].

### 3.3. Thermal Behavior

**Figure 5:**
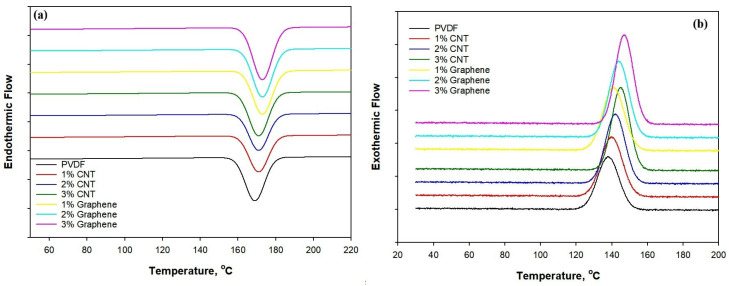
DSC thermogram of CNT/PVDF and Graphene/PVDF nanocomposite: (**a**) Heating; (**b**) cooling.

Figure 5 reveals the DSC heating (a) and cooling (b) curves of PVDF/CNT and Graphene/PVDF composites, while the corresponding data, including melting temperature, crystallization temperature and crystallinity, are shown in Table 1. The crystallinity was calculated by using the following Equation (3).

(3) $$X_{c}\;\%=\frac{∆H_{m}}{w\times \;∆H_{o}}\times 100$$

where *ΔH_m_* determines the melting enthalpy and *w* signifies the PVDF fraction of weight for the MWCNT and graphene-reinforced PVDF composite. As both α and β crystalline phases coexist in the PVDF nanofibers, the reference melting enthalpy (*ΔH_o_*) of fully crystalline PVDF was calculated using a weighted average of 38% α-phase (104.7 J g^−1^) and 62% β-phase (103.4 J g^−1^). The resulting reference enthalpy of approximately 103.9 J g^−1^ was then employed for the determination of the degree of crystallinity.

Figure 5a shows the DSC heating curves of neat PVDF and PVDF composites containing different loadings of CNTs and graphene, while Figure 5b presents the corresponding cooling curves. The results are shown in Table 2.

Neat PVDF exhibits a clear endothermic melting peak at 170.6 °C, confirming its semicrystalline nature. With the incorporation of CNTs and graphene, the melting temperature gradually shifts to higher values, reaching 174.5 °C for 3 wt.% CNT and 174.4 °C for 3 wt.% graphene. This increase in melting temperature indicates improved crystal perfection and enhanced thermal stability due to restricted polymer chain mobility arising from strong interfacial interactions between PVDF and the carbon nanofillers. During cooling, neat PVDF crystallizes at 139.2 °C, whereas the crystallization temperature increases progressively with increasing CNT and graphene content, attaining 144.1 °C and 144.8 °C for 3 wt.% CNT and 3 wt.% graphene, respectively. The upward shift in crystallization temperature demonstrates the effective role of CNTs and graphene as heterogeneous nucleating agents that promote earlier and faster crystallization of PVDF. Consequently, the degree of crystallinity increases from 47.0% for neat PVDF to 55.14% and 56.09% for 3 wt.% CNT and graphene-filled composites, respectively, with graphene showing a slightly stronger nucleation efficiency [46]. The heating cycle confirmed the thermal stability of these crystalline transitions, demonstrating that the conductive nanofillers not only influence the crystallization kinetics but also promote β-phase formation, an essential factor for improving the dielectric and electromagnetic shielding properties of the composite [47]. The DSC results reveal a well-balanced interplay between crystallinity, phase composition, and interfacial interactions, confirming the structural integrity and thermally stable nature of the CNT- and graphene-reinforced electrospun PVDF systems.

**Figure 6:**
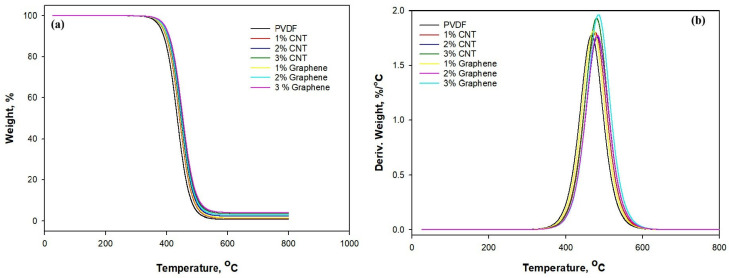
(**a**) TGA of CNT/PVDF and Graphene/PVDF; (**b**) DTG of CNT/PVDF and Graphene/PVDF.

Thermogravimetric analysis (TGA) of the electrospun PVDF systems is shown in Figure 6a,b. Table 3 reveals that the incorporation of carbonaceous nanofillers (CNTs and graphene) improves the thermal stability of the polymer matrix under an inert atmosphere.

Pure electrospun PVDF displays an onset temperature (Tonset) of rapid mass loss near 390 °C with a DTG peak of maximum temperature (Tmax) around 470 °C and leaves a negligible char of around 0.8% at 600 °C. The addition of CNTs produces a progressive stabilization, which shows that 1 wt.% CNT increases the apparent onset temperature to 400 °C and shifts the DTG maximum to 474 °C, while 3 wt.% CNT further raises Tonset to 410 °C and increases the residual mass to 3.5% at 600 °C. Graphene-filled samples follow a similar trend but exhibit slightly higher stabilization for the same filler content. Similarly, with 1% graphene, the onset temperature reaches 402 °C, and the maximum degradation temperature is 475 °C, accompanied by a residue of 1.5%. At 2% graphene, the onset temperature increases to 407 °C and the maximum degradation temperature rises to 482 °C, resulting in a higher residue of 2.8%. When the graphene loading is increased to 3 wt.%, the onset temperature and the maximum degradation temperature rise to approximately 412 °C and 486 °C, respectively, with a residue of 4.0%. This increase in both temperature and residue is consistent with graphene’s larger planar surface area and its stronger char-forming effect. The observed stabilization is attributable to a physical barrier effect where well-dispersed fillers hinder volatile decomposition-product diffusion and promote char formation that protects the underlying polymer from further thermal attack [48]. These results indicate that modest loadings (2–3 wt.%) of CNT and graphene meaningfully enhance the thermal validity of electrospun PVDF, which is an important advantage for applications requiring thermal endurance, such as EMI shielding in elevated temperatures.

### 3.4. Mechanical Characteristics

**Figure 7:**
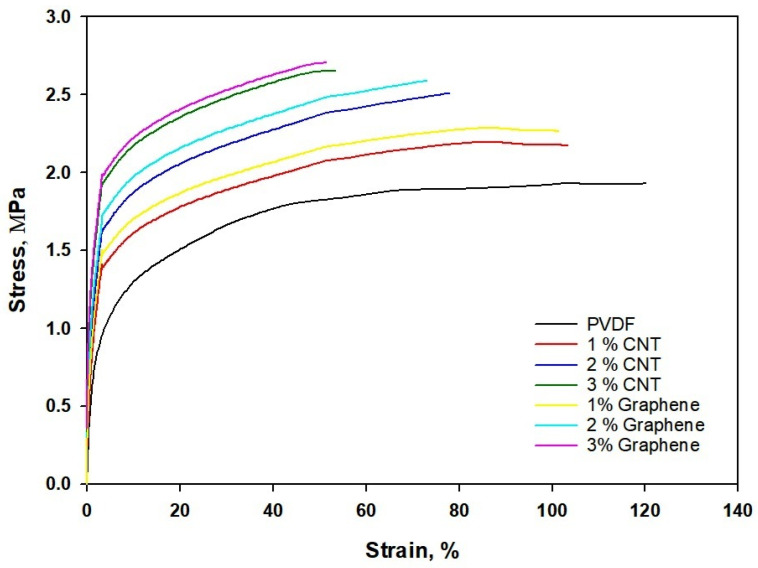
Stress–Strain behavior of CNT/PVDF and Graphene/PVDF nanocomposite.

Stress–strain behavior of electrospun CNT/PVDF and Graphene/PVDF is shown in Figure 7. Table 4 shows the mechanical properties of the electrospun mat with concentrations of MWCNT and graphene.

Pure PVDF exhibits a typical ductile polymeric behavior with a relatively low Young’s modulus (0.41 MPa) and ultimate tensile strength (UTS) of 1.81 MPa, but a high elongation at break of around 116%. The long polymer chains and low crystallinity allow substantial deformation before fracture, leading to a smooth and extended plastic region. When CNTs are incorporated, the stiffness and strength of the fibers increase progressively with filler content (1–3 wt.%), while ductility decreases. The CNTs serve as effective load-bearing nanofillers, improving interfacial stress transfer within the PVDF matrix. At 3 wt.% CNT loading, the modulus reaches 0.74 GPa, and the UTS increases to 2.61 MPa, though the elongation at break drops to 40%. This indicates that the nanofillers restrict chain mobility, promoting a more brittle fracture mode. Similarly, Graphene/PVDF composites display even higher stiffness and tensile strength at comparable filler loadings due to the planar structure and larger aspect ratio of graphene nanosheets. At 3 wt.%, Graphene/PVDF achieves an ultimate tensile strength of 2.65 MPa and modulus of 0.74 GPa, which is the highest among all samples with a corresponding elongation of 59%. The strong interfacial adhesion and efficient load transfer through graphene sheets enhance mechanical reinforcement.

### 3.5. Electrical Properties

**Figure 8:**
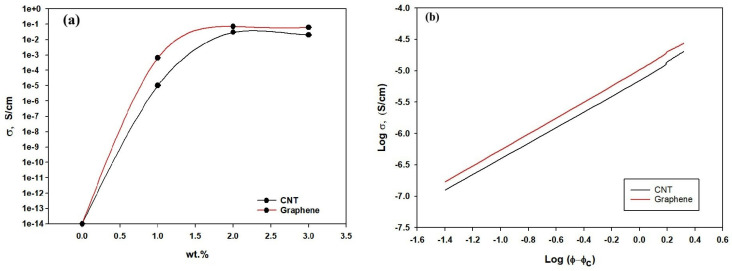
(**a**) Electrical conductivity of CNT/PVDF and Graphene/PVDF nanocomposite. (**b**) Percolation threshold fitting of electrospun CNT/PVDF and Graphene/PVDF nanocomposites.

The electrical behavior of the electrospun CNT/PVDF and Graphene/PVDF composite fibers is shown in Figure 8a. The obtained results demonstrate a clear structure–property relationship governed by the formation of conductive networks and phonon transport mechanisms. Pure PVDF displays very low DC conductivity (~10^−14^ ± 5.0 × 10^−16^ S/cm), characteristic of a highly insulating polymer matrix. At 1 wt.% CNT, a modest increase around 10^−5^ ± 6.0 × 10^−7^ S/cm is observed, indicating the presence of isolated conductive islands. When the filler loading reaches 2 wt.%, the conductivity increases by nearly three orders of magnitude, marking the percolation threshold where a partially connected CNT network allows limited charge transport [48]. At 3 wt.% CNT, a continuous conductive network forms throughout the PVDF matrix, resulting in a dramatic rise up to 0.02 ± 0.001 S/cm in DC conductivities reflecting the dominance of tunneling and hopping mechanisms across closely spaced CNTs. In the case of electrospun Graphene/PVDF composites, the conductivity enhancement follows a similar trend but with slightly higher DC conductivity values compared to CNT-filled samples at the same filler content [49]. This can be attributed to the two-dimensional geometry and large aspect ratio of graphene sheets, which facilitate interfacial charge transfer and electron delocalization along extended planar pathways. The incorporation of 1 wt.% and 2 wt.% graphene leads to a pronounced enhancement in DC electrical conductivity, reaching ~10^−4^ ± 5.0 × 10^−7^ S/cm and 10^−2^ ± 0.0011 S/cm, respectively. At 3 wt.% graphene, a fully developed network enables DC conductivity around 0.011 ± 0.001 S/cm, indicating efficient electron transport across overlapped graphene. Both CNT and graphene fillers significantly improve the electrical performance of electrospun PVDF fibers. The percolation-driven transition from insulating to semiconducting behavior confirms the formation of continuous conductive networks with graphene-based composites, demonstrating slightly superior performance due to their sheet-like morphology and stronger interfacial interactions.

In percolation theory, the electrical conductivity above the percolation threshold follows the power law relation as shown in Equation (4).

(4) $$\sigma =\sigma_{o}{\left(\varphi -\varphi_{C}\right)}^{t}\;for\;\varphi >\varphi_{C}$$

where *σ* is the DC electrical conductivity, *φ* is the filler loading, *φc* is the percolation threshold, and *t* is the critical exponent that reflects the dimensionality and efficiency of the conductive network.

Figure 8b demonstrates a linear dependence of log σ on (φ − φc), confirming percolation-controlled charge transport. It was evaluated that the electrospun CNT/PVDF exhibits a percolation threshold of approximately 0.9 wt.%. Similarly, the electrospun Graphene/PVDF demonstrates a lower percolation threshold of 0.7 wt.%, followed by a more rapid rise in conductivity due to the two-dimensional morphology and larger interfacial contact area of graphene sheets. The fitted conductivity data of the electrospun CNT/PVDF composites reveal a percolation exponent of approximately *t* ≈ 2.1, consistent with the formation of a three-dimensional rod-like conductive network. The relatively higher *t* value indicates that conductivity increases gradually after percolation, consistent with electron transport dominated by CNT–CNT junction resistance and tunneling across point contacts. In contrast, graphene/PVDF composites exhibit a lower percolation exponent of *t* ≈ 1.6, reflecting a more efficient conductive network formed by two-dimensional graphene sheets. The reduced *t* value signifies a steeper rise in conductivity above the percolation threshold, which is attributed to the larger contact area, lower junction resistance and enhanced charge carrier mobility provided by graphene platelets. These findings demonstrate that the percolation characteristics of the conductive network play a critical role in governing absorption-controlled EMI shielding.

### 3.6. Electromagnetic Shielding Interference

**Figure 9:**
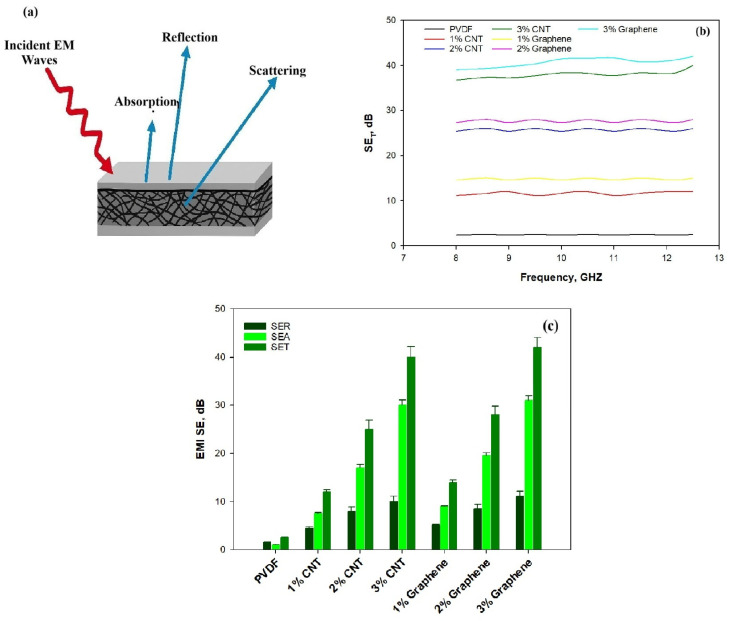
(**a**) Mechanism of EMI shielding. (**b**) SE_T_ of electrospun CNT/PVDF and Graphene/PVDF nanocomposites with respect to frequency. (**c**) Average values of SE_T_, SE_R_ and SE_A_ of electrospun CNT/PVDF and Graphene/PVDF nanocomposite.

Figure 9a shows the EMI shielding mechanism of electrospun CNT/PVDF and Graphene/PVDF composites. Upon electromagnetic wave incidence, the surface layer characterized by electrical conductivity and high magnetic loss enables efficient wave penetration and promotes enhanced absorption. Additionally, EMI shielding effectiveness (SE) is determined from the critical logarithmic ratio of the incoming power, Pin, to outgoing power, Pout. The total EMI shielding, SET, shielding of absorption, SEA, and shielding of reflection, SER, were achieved by recording most reflection coefficient (S11) parameters and transmission coefficients (S21) parameters. The EMI SET is calculated by using Equation (5).

(5) $${EMISE}_{T}={SE}_{R}+{SE}_{A}=10\log\left(\frac{P_{in}}{P_{out}}\right)\left(dB\right)$$

Figure 9b illustrates the variation in total electromagnetic shielding effectiveness (SE_T_) as a function of frequency (8–12.5 GHz) for neat PVDF and PVDF composites reinforced with carbon nanotubes (CNT) and graphene (Gr) at different filler loadings (1–3 wt.%). Neat PVDF exhibits very low SE_T_ (~2.5 dB), confirming its dielectric and non-conductive nature. Upon incorporation of conductive nanofillers, a significant enhancement in shielding performance is observed, with SE_T_ strongly dependent on filler type and loading. For the CNT/PVDF system, the SE_T_ increases from 12 dB at 1 wt.% CNT to 26 dB at 2 wt.% CNT and reaches 40 dB at 3 wt.% CNT, corresponding to an attenuation of more than 99.99% of incident electromagnetic radiation at the highest loading. This sharp enhancement reflects the progressive formation of a percolated CNT network, which facilitates charge transport, interfacial polarization, and multiple internal reflections within the porous fibrous structure, thereby promoting absorption-dominated EMI shielding. A similar but slightly superior shielding performance is observed for the Graphene/PVDF composites. The SE_T_ attains 15 dB at 1 wt.% graphene, increases markedly to 28 dB at 2 wt.%, and reaches 42 dB at 3 wt.% graphene. Graphene-reinforced composites exhibit higher SE_T_ than CNT-based composites at equivalent loadings. This superior performance is attributed to graphene’s two-dimensional sheet-like morphology, larger surface area and higher aspect ratio which enable the formation of denser conductive networks and provide extended pathways for electron transport [50]. The electromagnetic interference (EMI) shielding effectiveness (SE_T_) shows a pronounced and systematic increase with increasing conductive filler content for both CNT and graphene-reinforced composites, highlighting the efficiency of nanocarbon fillers in developing conductive shielding networks. The results demonstrate that graphene-based PVDF composites, particularly at 3 wt. % loading, are highly effective EMI shielding materials suitable for lightweight and flexible applications.

To elucidate the dominant shielding mechanism, Figure 9c, the total EMI shielding effectiveness (SET) was decomposed into contributions from reflection loss (SE_R_) and absorption loss (SE_A_), as shown in Figure 9c. SE_A_ consistently dominates over SE_R_ for both CNT- and graphene-filled PVDF composites, confirming an absorption-controlled shielding mechanism. This behavior is particularly evident at higher filler loadings (2–3 wt.%), where SE_A_ contributes the major fraction of SE_T_. Neat PVDF exhibited negligible shielding effectiveness, with average values of SE_R_ ≈ 1 dB, SE_A_ ≈ 1.5 dB, and SE_T_ ≈ 2.5 dB, reflecting its insulating nature. In contrast, the incorporation of conductive nanofillers led to a pronounced enhancement in EMI shielding. For CNT-reinforced PVDF, SE_T_ increased progressively from ~12 dB at 1 wt.% CNT (SE_R_ ≈ 4.5 dB, SE_A_ ≈ 7.5 dB) to ~26 dB at 2 wt.% CNT (SE_R_ ≈ 8 dB, SE_A_ ≈ 18 dB), reaching ~40 dB at 3 wt.% CNT, where SE_A_ (~30 dB) dominated over SE_R_ (~10 dB). A similar but more pronounced trend was observed for graphene-filled composites, which consistently outperformed CNT-based systems. At 1 wt.% graphene, SE_T_ reached ~14 dB (SE_R_ ≈ 5 dB, SE_A_ ≈ 9 dB), increasing to ~28 dB at 2 wt.% graphene (SE_R_≈ 8.5 dB, SE_A_ ≈ 19.5 dB) and achieving a maximum SE_T_ of 42 dB at 3 wt.% graphene, with SE_A_ (31 dB) as the dominant contribution. Additionally, the presence of CNTs and graphene creates numerous conductive junctions and heterogeneous interfaces, which facilitate dipolar polarization and Maxwell–Wagner–Sillars (MWS) interfacial polarization, thereby improving absorption efficiency [51,52,53]. The dominance of absorption-controlled shielding is attributed to enhanced electrical conductivity, reduced skin depth, interfacial polarization, and multiple internal scattering within the well-developed conductive networks [54]. Previously reported CNT/PVDF and Graphene/PVDF composites generally exhibit EMI shielding effectiveness in the range of 18–27 dB, depending on filler loading [31]. CNT/PVDF composites typically show shielding values of about 22 dB, while graphene/PVDF systems demonstrate around 18–19 dB [40]. The current study demonstrates the synergistic integration of CNTs and graphene within electrospun PVDF nanofibers to construct a conductive network for absorption-dominated EMI shielding that increases up to 40–42 dB. Both CNT and graphene-reinforced PVDF composites exhibit absorption-dominated EMI shielding behavior, making them attractive candidates for lightweight, flexible, and high-performance EMI shielding applications.

To assess the economic competitiveness of the developed materials with 3 wt.% CNT and graphene, the cost efficiency was therefore evaluated in terms of shielding effectiveness per unit area [55]. Assuming a composite thickness of *t* mm, a surface area of *A* m^2^, and a measured EMI shielding effectiveness (*SE*) of *X* dB, the cost per dB·m^2^ was calculated as follows (6):

(6) $${Cost}_{per\;dB\cdot m^{2}}=\frac{Materials\;cost\;per{\;m}^{2}}{EMISE\;(dB)}$$

Pure PVDF shows a relatively high cost per unit shielding effectiveness of ~1.44 USD/(dB·m^2^) as a result of its low SE_T_ (~2.5 dB), underscoring the economic inefficiency of the neat polymer. In contrast, electrospun PVDF mats containing 3 wt.% CNTs exhibit a markedly reduced cost effectiveness of ~0.10 USD/(dB·m^2^) while graphene-filled PVDF at the same loading achieves an even lower value of ~0.09 USD/(dB·m^2^), demonstrating substantially enhanced shielding performance per unit cost.

## 4. Conclusions

In this work, flexible and lightweight electrospun PVDF-based nanocomposites incorporating carbon nanotubes (CNTs) and graphene were successfully developed and systematically evaluated for electromagnetic interference (EMI) shielding and multifunctional performance. Structural and spectroscopic analyses confirmed that the incorporation of CNTs and graphene did not alter the chemical backbone of PVDF but significantly promoted the formation of the electroactive β-phase, which is beneficial for electrical polarization and electromagnetic attenuation. Thermal analyses demonstrated that the nanofillers improved the crystallization behavior and thermal stability of PVDF, indicating strong interfacial interactions and enhanced resistance to thermal degradation. Mechanical and morphological investigations revealed that the electrospun fibrous architecture, combined with well-dispersed conductive fillers, produced mechanically robust and flexible mats suitable for practical applications. Electrical conductivity measurements showed a clear percolation-driven enhancement with increasing CNT and graphene content, which directly translated into superior EMI shielding performance. In the X-band frequency range (8.0–12.5 GHz), the CNT/PVDF and Graphene/PVDF composites exhibited absorption-dominated shielding behavior, with total shielding effectiveness (SET) reaching ~40 dB for CNT/PVDF (3 wt.%) and up to ~42 dB for Graphene/PVDF (3 wt.%). The results demonstrate that electrospun CNT/PVDF and Graphene/PVDF nanocomposites combine effective EMI shielding, mechanical flexibility and thermal stability within a single material platform. These attributes position the developed composites as promising candidates for next-generation EMI shielding applications in biomedical devices, wearable electronics, and flexible electronic systems, where lightweight, absorption-dominated, and biocompatible materials are critically required.

## Figures and Tables

**Table 1:** Process parameters for PVDF/MWCNT and PVDF/Graphene electrospun.

Sample	Solvent Mixture DMF/Acetone (g)	PVDF (g)	MWCNT (g)	Feed Rate (mL/h)	Needle to Collector Distance (cm)	Voltage (kV)
PVDF	13.0000	2.7500	-	0.8	15	15
1% MWCNT	13.0000	2.7512	0.0155	0.8	15	15
2% MWCNT	13.0000	2.7513	0.0331	0.8	15	16
3% MWCNT	13.0000	2.7521	0.0594	0.8	15	17
1% Graphene	13.0000	2.7513	0.0154	0.8	15	16
2% Graphene	13.0000	2.7513	0.0331	0.8	15	17
3% Graphene	13.0000	2.7513	0.0594	0.8	15	18

**Table 2:** Thermal properties of CNT and graphene-reinforced electrospun PVDF.

Sample	Tm (°C)	Tc (°C)	X_c_, %
PVDF	170.6	139.2	47.0
1% CNT	171.2	139.7	50.13
2% CNT	172.3	141.4	53.09
3% CNT	174.5	144.1	55.14
1% Graphene	171.8	140.3	51.12
2% Graphene	173.0	142.6	53.97
3% Graphene	174.4	144.8	56.09

**Table 3:** Thermogravimetric analysis (TGA) results.

Sample	Tonset (°C)	Tmax (°C) (DTG Peak)	Residue @600 °C (%)
PVDF	390	470	0.8
1% CNT	400	474	1.3
2% CNT	405	482	2.2
3% CT	410	486	3.5
1% Graphene	402	475	1.5
2% Graphene	407	482	2.8
3% Graphene	412	486	4.0

**Table 4:** Mechanical properties of the electrospun mat.

Sample	Tensile Strength, MPa	Elastic Modulus, GPa	Elongation at Break, %
PVDF	1.8 ± 0.11	0.41 ± 0.03	116.12 ± 0.20
1% CNT	2.38 ± 0.18	0.56 ± 0.02	90.33 ± 0.15
2% CNT	2.50 ± 0.14	0.66 ± 0.4	75.95 ± 0.18
3% CT	2.61 ± 0.14	0.74 ± 0.3	60.45 ± 0.17
1% Graphene	2.42 ± 0.13	0.57 ± 0.04	89.1 ± 0.15
2% Graphene	2.53 ± 0.14	0.68 ± 0.03	73.2 ± 0.19
3% Graphene	2.65 ± 0.15	0.74 ± 0.02	59.4 ± 0.14

## Data Availability

The original contributions presented in this study are included in the article. Further inquiries can be directed to the corresponding author.
